# An essential role for neuregulin-4 in the growth and elaboration of developing neocortical pyramidal dendrites

**DOI:** 10.1016/j.expneurol.2018.01.002

**Published:** 2018-04

**Authors:** Blanca Paramo, Sean Wyatt, Alun M. Davies

**Affiliations:** School of Biosciences, Cardiff University, Museum Avenue, Cardiff CF10 3AX, Wales, UK

**Keywords:** Neuregulin, Cortical pyramidal neuron, Dendrite, Knockout mouse, Development

## Abstract

Neuregulins, with the exception of neuregulin-4 (NRG4), have been shown to be extensively involved in many aspects of neural development and function and are implicated in several neurological disorders, including schizophrenia, depression and bipolar disorder. Here we provide the first evidence that NRG4 has a crucial function in the developing brain. We show that both the apical and basal dendrites of neocortical pyramidal neurons are markedly stunted in *Nrg4*^−/−^ neonates *in vivo* compared with *Nrg4*^+/+^ littermates. Neocortical pyramidal neurons cultured from *Nrg4*^−/−^ embryos had significantly shorter and less branched neurites than those cultured from *Nrg4*^+/+^ littermates. Recombinant NRG4 rescued the stunted phenotype of embryonic neocortical pyramidal neurons cultured from *Nrg4*^−/−^ mice. The majority of cultured wild type embryonic cortical pyramidal neurons co-expressed NRG4 and its receptor ErbB4. The difference between neocortical pyramidal dendrites of *Nrg4*^−/−^ and *Nrg4*^+/+^ mice was less pronounced, though still significant, in juvenile mice. However, by adult stages, the pyramidal dendrite arbors of *Nrg4*^−/−^ and *Nrg4*^+/+^ mice were similar, suggesting that compensatory changes in *Nrg4*^−/−^ mice occur with age. Our findings show that NRG4 is a major novel regulator of dendritic arborisation in the developing cerebral cortex and suggest that it exerts its effects by an autocrine/paracrine mechanism.

## Highlights

• First evidence that neuregulin-4 (NRG4) functions in the nervous system.
• Neocortical pyramidal dendrites are markedly stunted in *Nrg4*^−/−^ neonates.
• Compensatory changes occur in the pyramidal dendrites of *Nrg4*^−/−^ mice with age.
• Neurons cultured from *Nrg4*^−/−^ embryos replicated the *in vivo* phenotype.
• Recombinant NRG4 rescued the stunted dendrite phenotype of these neurons.

## 1 Introduction

The neuregulins are widely expressed pleiotropic growth factors related to epidermal growth factor that signal *via* the ErbB family of receptor tyrosine kinases (Britsch, 2007). Very extensive work on the first neuregulin discovered (NRG1), has revealed that its numerous isoforms play many roles in the development and function of neurons and glia, including regulating the assembly of neural circuitry, myelination, neurotransmission and synaptic plasticity (Mei and Nave, 2014). Likewise, numerous studies on NRG2 and NRG3 have revealed that they participate in synaptogenesis, synaptic function and aspects of neuronal development (Bartolini et al., 2017, Lee et al., 2015, Vullhorst et al., 2015). Importantly, the *Nrg1*, *Nrg2*, *Nrg3*, *ErbB3* and *ErbB4* genes have been identified as susceptibility genes for schizophrenia, depression and bipolar disorder (Mei and Nave, 2014, Rico and Marin, 2011) and numerous genetic and functional studies have directly implicated the *Nrg1*, *Nrg2*, *Nrg3* and *ErbB4* genes in the development of psychotic behaviour (Hayes et al., 2016, O'Tuathaigh et al., 2007, O'Tuathaigh et al., 2010, Shamir et al., 2012, Yan et al., 2017). Although much less work has been done on the latest neuregulins to be identified, NRG5 and NRG6, both are highly expressed in brain (Kanemoto et al., 2001, Kinugasa et al., 2004). NR6 plays a role in radial neuronal migration in the neocortex (Zhang et al., 2013) and is a potential susceptibility gene for schizophrenia (So et al., 2010).

In contrast with other neuregulins, NRG4 is expressed in a limited number of adult tissues, such as brown adipose tissue, and has been reported to have no or negligible expression in adult brain (Harari et al., 1999, Rosell et al., 2014). NRG4 functions as a secreted endocrine factor *in vivo* produced and released by brown adipose tissue. NRG4 decreases hepatic lipogenesis, increases fatty acid β-oxidation and increases energy expenditure (Chen et al., 2017, Wang et al., 2014). While NRG4 has been implicated in the regulation of metabolic homeostasis, it has no known function in the brain. Our analysis of mice in which the *Nrg4* locus has been disrupted reveals a very striking phenotype in neocortical pyramidal neurons both *in vitro* and *in vivo*. As such, we provide the first evidence that NRG4 plays a major role in the brain.

## 2 Materials and methods

### 2.1 Animals

Mice were housed in a 12 h light-dark cycle with access to food and water *ad libitum*. Breeding was approved by the Cardiff University Ethical Review Board and was performed within the guidelines of the Home Office Animals (Scientific Procedures) Act, 1986. *Nrg4* null mice in which the *Nrg4* locus was disrupted by retroviral insertion of a gene trap between exons 1 and 2 were purchased from the Mutant Mouse Resource Centre, UC Davis (California, USA). These mice were backcrossed from a C57/BL6 background into a CD1 background. *Nrg4*^+/−^ mice were crossed to generate *Nrg4*^+/+^ and *Nrg4*^−/−^ littermates.

### 2.2 Neuron culture

Primary cortical neurons were prepared from E16 embryos. The protocol for culturing hippocampal pyramidal neurons was used with modifications (Kaech and Banker, 2006). Briefly, dissected cortices were mechanically triturated in Neurobasal A medium supplemented with 2% B27 (Gibco), 0.5 mM GlutaMAX 1, 100 units/ml penicillin and 100 μg/ml streptomycin (Invitrogen). 15,000 cells/cm^2^ were plated on poly-l-Lysine (Sigma)-coated 35 mm dishes and incubated at 37 °C in a humidified atmosphere with 5% CO_2_. In some cultures, the culture medium was supplemented with 100 ng/ml recombinant NRG4 (Thermo Fisher Scientific) after plating. Neurons were cultured for either 3 or 9 days *in vitro*. Neurons were fluorescently labelled in 3 day cultures by treating the cultures with the fluorescent dye calcein-AM (2 μg/ml, Invitrogen) for 15 min at 37 °C. In 9 days cultures, the neurite arbors of a subset of the neurons were visualized by transfecting the neurons with a GFP expression plasmid using lipofectamine 2000 (Invitrogen, Paisley, UK) after 7 days *in vitro*. Briefly, 1 μg of DNA was mixed with 2 μl of lipofectamine. After 20 min, this mixture in 2 ml of Opti-MEM media (Gibco) was added to the cultures. After 3 h at 37 °C, the cultures were washed with culture medium and incubated for a further 2 days. At the end of the experiment, the neurons were fixed for 30 min with 4% paraformaldehyde. Images of fluorescent-labelled neurons were acquired with an Axiovert 200 Zeiss fluorescent microscope. Neurite length and Sholl analysis were carried out using Fiji (ImageJ) software with the semi-automated plugin Simple Neurite Tracer (Longair et al., 2011).

### 2.3 Immunohistochemistry

Brains were fixed overnight using 4% paraformaldehyde in 0.12 M phosphate-buffered saline (PBS) at 4 °C, washed in PBS and cryoprotected in 30% sucrose before being frozen in dry ice-cooled isopentane. Serial 30 μm sections were blocked in 1% BSA (Sigma), 0.1% Triton (Sigma) in PBS and then incubated with 1:500 anti-MAP2 (Millipore) and anti-NRG4 (Abcam) antibodies at 4 °C overnight. After washing, the sections were incubated with 1:500 rabbit polyclonal Alexa-conjugated secondary antibodies (Invitrogen) for 1 h at room temperature. Sections were washed, incubated with DAPI and visualized using a Zeiss LSM710 confocal microscope.

### 2.4 Immunocytochemistry

Neurons were fixed for 10 mins in 4% paraformaldehyde in 0.12 M phosphate-buffered saline (PBS), washed 3 times in PBS and blocked in 1% BSA (Sigma), 0.1% Triton (Sigma) in PBS for 1 h, then incubated with primary antibodies (1:50) against NRG4 (Santa Cruz), ErbB4 (Abcam), Emx1 (Santa Cruz) overnight at 4 °C. After washing, the neurons were incubated with polyclonal Alexa-conjugated secondary antibodies (Invitrogen) 1:500 for 1 h at room temperature. Cells were then washed, incubated with DAPI (1:8000) and visualized using a Zeiss LSM710 confocal microscope.

### 2.5 Quantitative PCR

The levels of *Nrg4* mRNA was quantified by RT-qPCR relative to a geometric mean of mRNAs for the house keeping enzymes glyceraldehyde phosphate dehydrogenase (*Gapdh*), succinate dehydrogenase (*Sdha*) and hypoxanthine phosphoribosyltransferase-1 (*Hprt1*). Total RNA was extracted from dissected tissues with the RNeasy Mini Lipid extraction kit (Qiagen, Crawely, UK). 5 μl total RNA was reverse transcribed, for 1 h at 45 °C, using the Affinity Script kit (Agilent, Berkshire, UK) in a 25 μl reaction according to the manufacturer's instructions. 2 μl of cDNA was amplified in a 20 μl reaction volume using Brilliant III ultrafast qPCR master mix reagents (Agilent Technologies). PCR products were detected using dual-labelled (FAM/BHQ1) hybridization probes specific to each of the cDNAs (MWG/Eurofins, Ebersberg, Germany). The PCR primers were: *Nrg4* forward: 5′-GAG ACA AAC AAT ACC AGA AC-3′ and reverse: 5′-GGA CTG CCA TAG AAA TGA-3′; *ErbB4* forward: 5′-GGC AAT ATC TAC ATC ACT G-3′ and reverse: 5′-CCA ACA ACC ATC ATT TGA A-3′; *Gapdh* forward: 5′-GAG AAA CCT GCC AAG TAT G-3′ and reverse: 5′-GGA GTT GCT GTT GAA GTC-3′; *Sdha* forward: 5′-GGA ACA CTC CAA AAA CAG-3′ and reverse: 5′-CCA CAG CAT CAA ATT CAT-3′; *Hprt1* forward: 5′-TTA AGC AGT ACA GCC CCA AAA TG-3′ and reverse: 5′-AAG TCT GGC CTG TAT CCA ACA C-3′. Dual-labelled probes were: *Nrg4*: 5′-FAM-CGT CAC AGC CAC AGA GAA CAC-BHQ1–3′; *ErbB4*: 5′-FAM-AGC AAC CTG TGT TAT TAC CAT ACC ATT-BHQ1–3′; *Gapdh*: 5′-FAM-AGA CAA CCT GGT CCT CAG TGT-BHQ1–3; *Sdha*: 5′-FAM-CCT GCG GCT TTC ACT TCT CT-BHQ1–3, *Hrpt1*: 5′-FAM-TCG AGA GGT CCT TTT CAC CAG CAA G-BHQ1–3′. Forward and reverse primers were used at a concentration of 250 nM and dual-labelled probes were used at a concentration of 500 nM. PCR was performed using the Mx3000P platform (Agilent) using the following conditions: 95 °C for 3 min followed by 45 cycles of 95 °C for 10 s and 60 °C for 35 s. Standard curves were generated for each cDNA for every real time PCR run, by using serial threefold dilutions of reverse transcribed adult mouse brain total RNA (Zyagen, San Diego, USA). Relative mRNA levels were quantified in whole brain, BAT and various brain regions dissected from at least 3 animals at each age. Primer and probe sequences were designed using Beacon Designer software (Premier Biosoft, Palo Alto, USA).

### 2.6 Golgi staining

Modified Golgi-Cox impregnation of neurons was performed using the FD Rapid GolgiStain kit (FD NeuroTechnologies) according to the manufacturer's instructions on 150 μm transverse sections of P10, P30 and adult (P60 and P70) brains of *Nrg4*^+/+^ and *Nrg4*^−/−^ mice. Total dendrite length, branch point number and Sholl analysis was carried out separately on the apical and basal dendrite compartments of cortical pyramidal neurons of P10 and P30 littermates using the plugin Sholl Analysis of Fiji software (Schindelin et al., 2012) after neuronal reconstruction with the plugin Simple Neurite Tracer (Longair et al., 2011).

## 3 Results and discussion

### 3.1 Expression of Nrg4 transcripts in developing brain

**Fig. 1:**
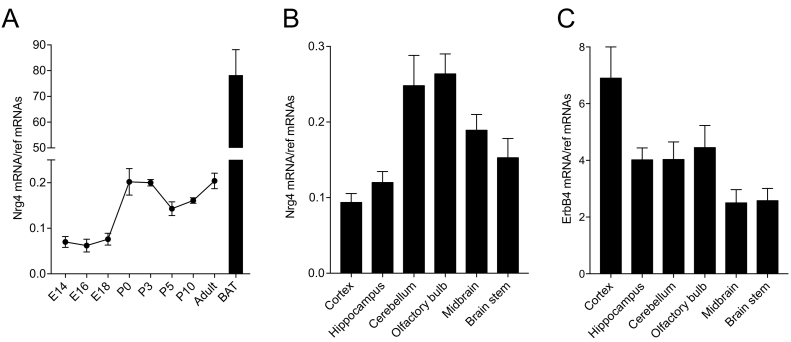
*Nrg4* mRNA is expressed in the developing brain. Levels of *Nrg4* mRNA in the neocortex at different ages compared with adult brown adipose tissue (BAT) (A) and in different brain regions at P0 (B) and the levels of *ErbB4* mRNA in these regions (C) relative to the geometric mean of reference mRNAs. The mean ± s.e.m. of data from four separate sets of tissues at each age/region are plotted. (For interpretation of the references to colour in this figure legend, the reader is referred to the web version of this article.)

To begin to explore the possibility that NRG4 functions in the developing brain, we used qPCR to investigate if *Nrg4* mRNA is expressed in the embryonic mouse neocortex. This revealed that *Nrg4* mRNA is clearly detectable in the embryonic neocortex, although its level is some 400-fold lower than that in adult brown adipose tissue. During development, there is an approximate 3-fold increase between E14 and birth (Fig. 1A). Measurement of *Nrg4* mRNA in newborn brain regions revealed that *Nrg4* mRNA is widely expressed, with the highest levels in the cerebellum and olfactory bulb (Fig. 1B). Measurement of *ErbB4* mRNA, which encodes the receptor for most neuregulins, including NRG4, revealed that this is also widely expressed in the newborn brain, with the highest levels in the neocortex (Fig. 1C).

### 3.2 Neocortical pyramidal dendrites are markedly stunted in neonatal Nrg4 −/− mice

**Fig. 2:**
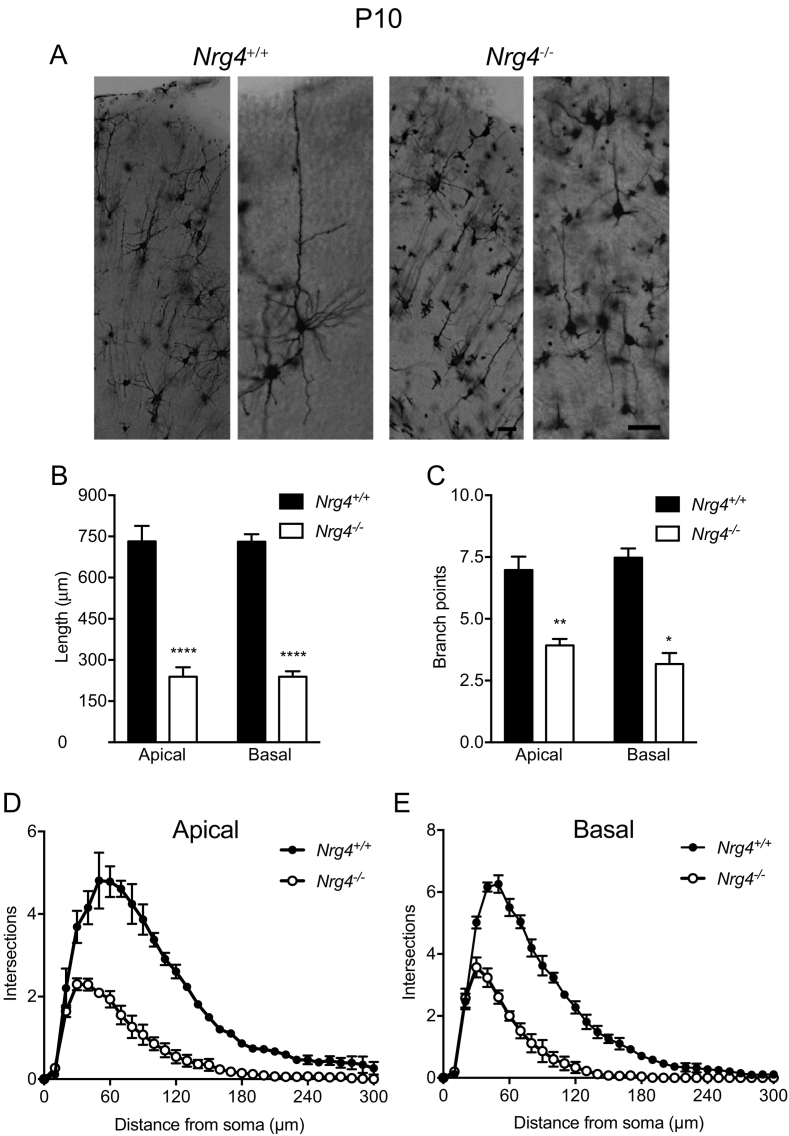
Phenotypic changes in neocortical pyramidal neurons of *Nrg4*^−/−^ P10 mice. (A) Representative low-power and high-power images of Golgi preparations of the motor neocortex of P10 *Nrg4*^+/+^ and *Nrg4*^−/−^ mice. Scale bar = 50 μm. Quantification of total dendrite length (B) and number of branch points (C) in the apical and basal dendrite compartments of pyramidal neurons in the motor neocortices of P10 *Nrg4*^+/+^ and *Nrg4*^−/−^ littermates. Sholl plots of the apical (D) and basal (E) dendrite compartments of these neurons. The mean ± s.e.m. of data from 90 neurons per genotype are plotted (*P < 0.05, **P < 0.01, ****P < 0.0001, statistical comparison between *Nrg4*^+/+^ and *Nrg4*^−/−^ mice, two-tailed *t*-test).

To investigate the significance of *Ngr4* mRNA expression in the developing brain, we compared the brains of *Nrg4*^−/−^ and *Nrg4*^+/+^ mice. Golgi preparations of transverse sections were made of the most rostral part of the neocortex, including the frontal/motor cortex and the most rostral region of the somatosensory cortex of postnatal day 10 (P10) mice. These revealed that the size and complexity of the dendritic arbors of pyramidal neurons throughout the full thickness of the neocortex were dramatically reduced in *Nrg4*^−/−^ mice compared with *Nrg4*^+/+^ littermates. Representative high power images show that the reduction of dendrite size and complexity in *Nrg4*^−/−^ mice affected both apical and basal dendrite compartments of these neurons (Fig. 2A). Analysis carried out separately on the apical and basal dendrite compartments revealed a highly significant four-fold reduction in total dendrite length (Fig. 2B) and a significant two-fold reduction in the number of branch points (Fig. 2C) in both dendrite compartments. Reductions in dendrite length and branching were reflected in the Sholl analyses of apical (Fig. 2D) and basal (Fig. 2E) compartments. These findings suggest that NRG4 plays a major and unexpected role in promoting the growth and elaboration of pyramidal neuron dendrites in the developing neocortex.

### 3.3 The pyramidal dendrite phenotype in Nrg4 −/− mice becomes less marked with age

**Fig. 3:**
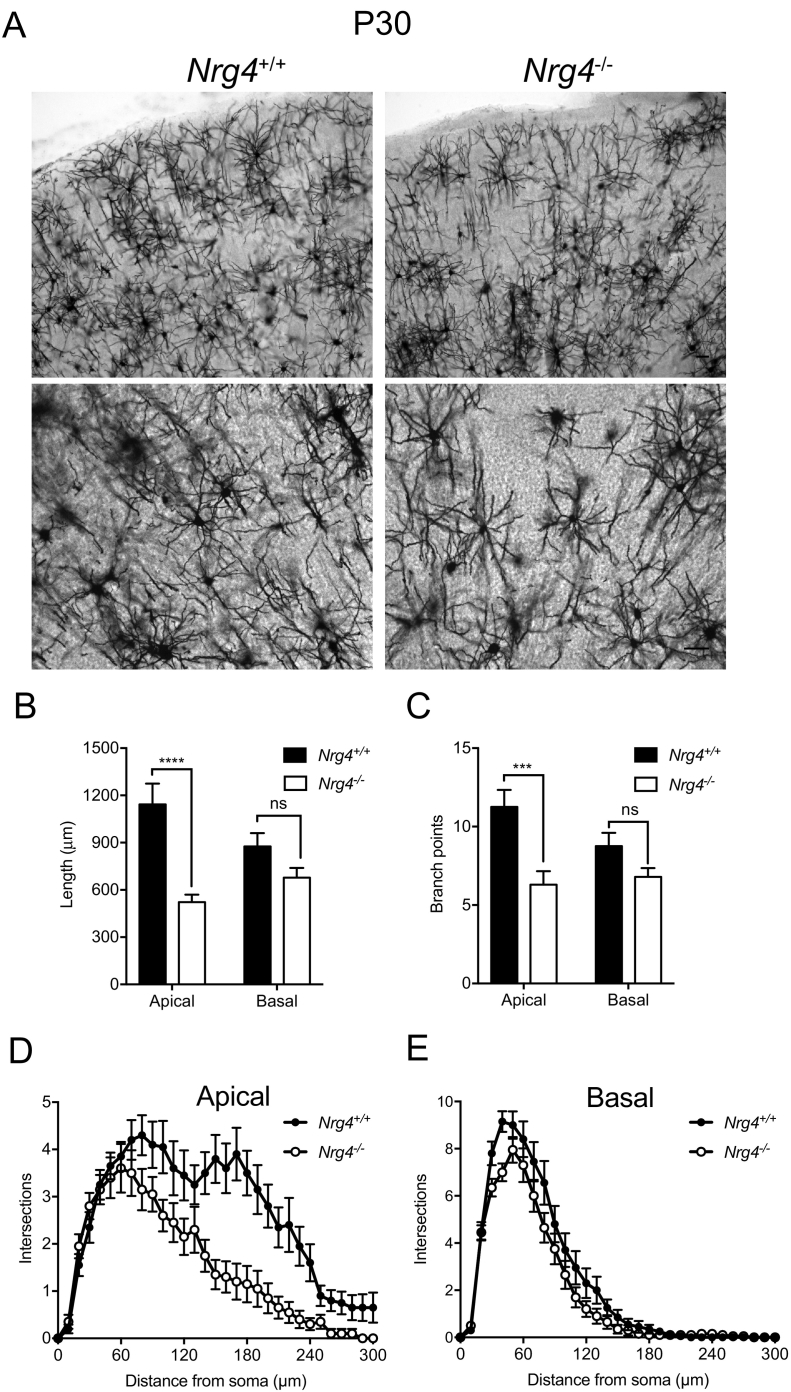
Phenotypic changes in neocortical pyramidal neurons of *Nrg4*^−/−^ P30 mice. (A) Representative low-power and high-power images of Golgi preparations of the motor neocortex of P30 *Nrg4*^+/+^ and *Nrg4*^−/−^ mice. Scale bar = 50 μm. Quantification of total dendrite length (B) and number of branch points (C) in the apical and basal dendrite compartments of pyramidal neurons in the motor neocortices of P30 *Nrg4*^+/+^ and *Nrg4*^−/−^ littermates. Sholl plots of the apical (D) and basal (E) dendrite compartments of these neurons. The mean ± s.e.m. of data from 20 neurons per genotype are plotted (ns, not significant, ***P < 0.001, ****P < 0.0001, statistical comparison between *Nrg4*^+/+^ and *Nrg4*^−/−^ mice, two-tailed t-test).

**Fig. 4:**
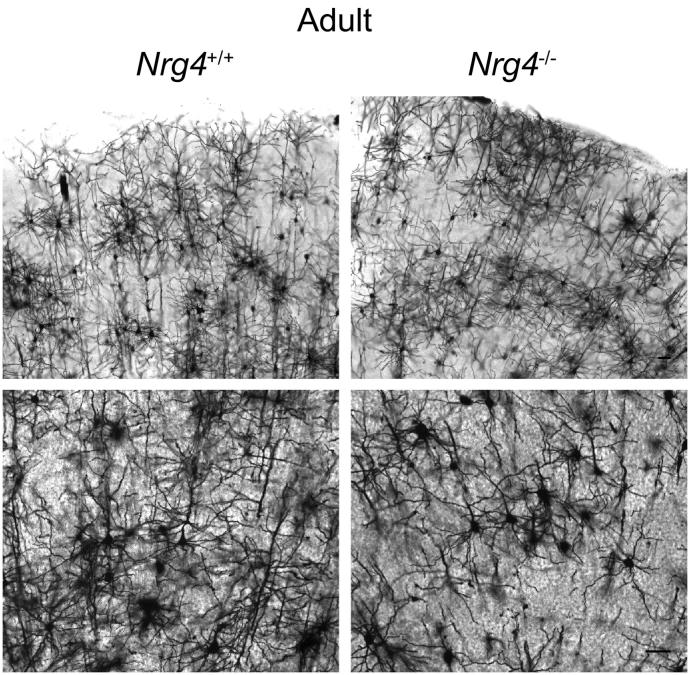
Neocortical pyramidal neurons of *Nrg4*^−/−^ and *Nrg4*^+/+^ adult mice. Representative low-power and high-power images of Golgi preparations of the motor neocortex of adult (P60 and P70) *Nrg4*^+/+^ and *Nrg4*^−/−^ mice. Four cortices of each genotype were examined (3 P60 *Nrg4*^+/+^, 3 P60 *Nrg4*^−/−^, 1 P70 *Nrg4*^+/+^, 1 P70 *Nrg4*^−/−^). Scale bar = 50 μm.

To determine whether the pronounced phenotype observed in neonatal *Nrg4*^−/−^ mice is retained into adulthood or whether compensatory changes occur with age, we repeated the Golgi studies in juvenile (P30) and adult (P60 and P70) littermates. In P30 mice, the difference in the size and complexity of neocortical pyramidal dendrites between *Nrg4*^+/+^ and *Nrg4*^−/−^ mice was still evident, though less pronounced than in neonates (Fig. 3A). Quantification confirmed this impression, and showed that both the apical and basal dendrites of neocortical pyramidal neurons of P30 *Nrg4*^−/−^ mice were shorter and less branched than those of *Nrg4*^+/+^ littermates (Fig. 3B and C). Compared with P10 preparations, the proportional differences in dendrite length and branching between *Nrg4*^−/−^ and *Nrg4*^+/+^ mice were less pronounced. While the differences were still highly significant for apical dendrites, they had lost statistical significance for basal dendrites by this age. The differences in apical and basal dendrite length and branching between *Nrg4*^−/−^ and *Nrg4*^+/+^ mice were reflected in the Sholl analyses of apical (Fig. 3D) and basal (Fig. 3E) compartments. In adult mice (P60 and P70) there did not appear to be pronounced differences in the size and complexity of neocortical pyramidal dendrite arbors of *Nrg4*^−/−^ and *Nrg4*^+/+^ mice (Fig. 4). However, the large size and complexity of neocortical pyramidal dendrites in adults precluded accurate quantification. Taken together, these findings suggest that compensatory changes in the pyramidal dendrites of *Nrg4*^−/−^ mice occur with age. This may be mediated by other members of neuregulin family, which are expressed at high levels in the mature brain.

Various phenotypic changes have been reported in neurons in genetic studies of other neuregulins and ErbB receptors. Although constitutive deletion of the *Nrg1*, *ErbB2* and *ErbB4* genes results in embryonic lethality, conditional deletion of *ErbB2/B4* in the brain decreases pyramidal neuron dendritic spine maturation in the cortex and hippocampus without affecting the gross dendrite morphology (Barros et al., 2009). Spine density is significantly reduced in cortical pyramidal neurons when *ErbB4* is conditionally deleted in these neurons (Cooper and Koleske, 2014) and synaptic spine size and density is likewise reduced in CA1 hippocampal pyramidal neurons by RNAi knockdown of *ErbB4* in these neurons (Li et al., 2007). While impaired dendritic spine formation has not been consistently observed following *ErbB4* deletion in pyramidal neurons (Fazzari et al., 2010, Yin et al., 2013), deletion of *ErbB4* in parvalbumin-positive GABAergic interneurons results in reduced spine density on hippocampal pyramidal neurons (Yin et al., 2013). Decreased dendritic spine density and impaired growth and elaboration of the basal dendrites of cortical pyramidal neurons have been reported in mice with targeted disruption of type III *Nrg1* (Chen et al., 2008, Chen et al., 2010a). Retroviral knockdown of *Nrg2* in granule cells impairs dendrite growth and branching from these neurons *in vivo* (Lee et al., 2015).

As well as interfering with the growth and elaboration of cortical pyramidal dendrites, in further work it will be interesting to ascertain whether deletion of *Nrg4* affects dendrite spine density and maturation and the functional properties of synapses. Moreover, we focused on neocortical pyramidal neurons in our current study. Given the widespread expression of *Nrg4* mRNA in multiple brain regions in the brain of newborns, it will be interesting to explore the consequences of *Nrg4* deletion more widely in the developing brain.

### 3.4 Neocortical neurons cultured from Nrg4^−/−^ embryos have stunted dendrites and axons

To ascertain whether the dendrite phenotype observed in developing *Nrg4*^−/−^ mice *in vivo* is replicated *in vitro* and can be rescued by NRG4 treatment, we set up dissociated neocortical cultures from *Nrg4*^−/−^ and *Nrg4*^+/+^ littermates. Cultures were established from the E16 cerebral cortex, a stage at which the predominant neuron type in culture is the pyramidal neuron, as shown by staining our cultures for Emx1, a homeodomain protein that is specifically expressed by pyramidal neurons in the developing cerebral cortex (Chan et al., 2001). > 80% cells in our cultures were positive for Emx1 after 3 days in culture. An added advantage of studying cultured pyramidal neurons is that dendrites and axons can be distinguished and studied separately (Banker and Cowan, 1979, Dotti et al., 1988, Kaech and Banker, 2006). After 3 days in culture, the single long axon that emerges from these neurons is clearly distinguishable from the multiple, short dendrites. After 9 days in culture, the axon remains the longest process and MAP-2-positive dendrites become well-developed.

**Fig. 5:**
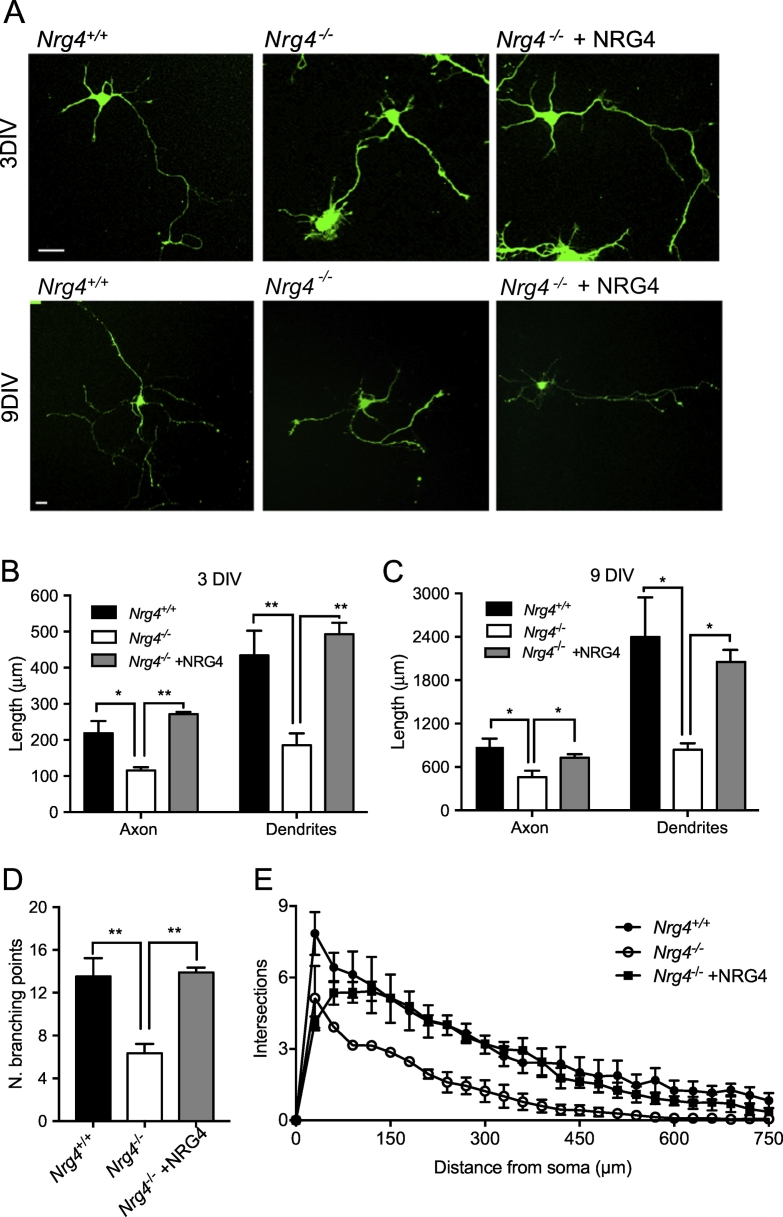
*Nrg4* enhances dendrite growth from cultured cortical pyramidal neurons. (A) Representative photomicrographs of cortical neurons of *Nrg4*^+/+^ and *Nrg4*^−/−^ E16 embryos cultured for either 3 or 9 days in culture. Neurons from *Nrg4*^−/−^ embryos were additionally treated with recombinant 100 ng/ml NRG4. Scale bar = 25 μm. Quantification of total neurite length in these cultures after either 3 days (B) or 9 days (C) *in vitro* and the number of branch points after 9 days *in vitro* (D). Sholl plots of neurons after 9 days *in vitro* (E). Mean ± s.e.m. of data collected from three independent experiments, *p < 0.01, and **p < 0.0001, one-way ANOVA with Holme-Sidak's multiple comparison post-hoc test.

Pyramidal neurons cultured from *Nrg4*^−/−^ mice were clearly smaller and less branched compared with those cultured from *Nrg4*^+/+^ littermates in both 3 day and 9 day cultures (Fig. 5A). Quantification of axon length and total dendrite length in the arbors of individual pyramidal neurons revealed significant reductions in axon and dendrite length in neurons cultured from *Nrg4*^−/−^ mice compared with those cultured from *Nrg4*^+/+^ littermates (Fig. 5B and C). Quantification of the number of branch points in the dendrite arbors of 9 day cultures also revealed a significant reduction in cultures established from *Nrg4*^−/−^ mice compared with *Nrg4*^+/+^ littermates (Fig. 5D). The differences in the size and complexity of neurite arbors of neurons cultured from *Nrg4*^−/−^ and *Nrg4*^+/+^ littermates were reflected in the Sholl plots of these neurons (Fig. 5E), which provide graphic illustrations of neurite length and branching with distance from the cell body. The reductions in axon and dendrite length and dendrite branching in pyramidal neurons cultured from *Nrg4*^−/−^ mice were completely restored to wild type levels by treatment with recombinant NRG4 (Fig. 5).

These results show that the pyramidal neuron phenotype observed *in vivo* in NRG4-deficient mice is replicated in cultured neurons and is rescued by soluble NRG4. Our findings also raise the possibility that NRG4 plays a role in regulating the growth of pyramidal axons as well as being required for the growth and branching of pyramidal dendrites. However, because the full extent of pyramidal neuron axons cannot be reliably discerned in Golgi preparations, we cannot definitively conclude that NRG4 enhances the growth of pyramidal axons *in vivo*. Although we have shown that recombinant NRG4 rescues the stunted phenotype of neocortical pyramidal neurons cultured from *Nrg4*^−/−^ mice, we cannot exclude the possibility that the metabolic changes that occur in these mice (Chen et al., 2017, Wang et al., 2014) contributes to the development of the *in vivo* phenotype that we observed in the neocortex.

### 3.5 Neocortical pyramidal neurons co-express NRG4 and ErbB4

**Fig. 6:**
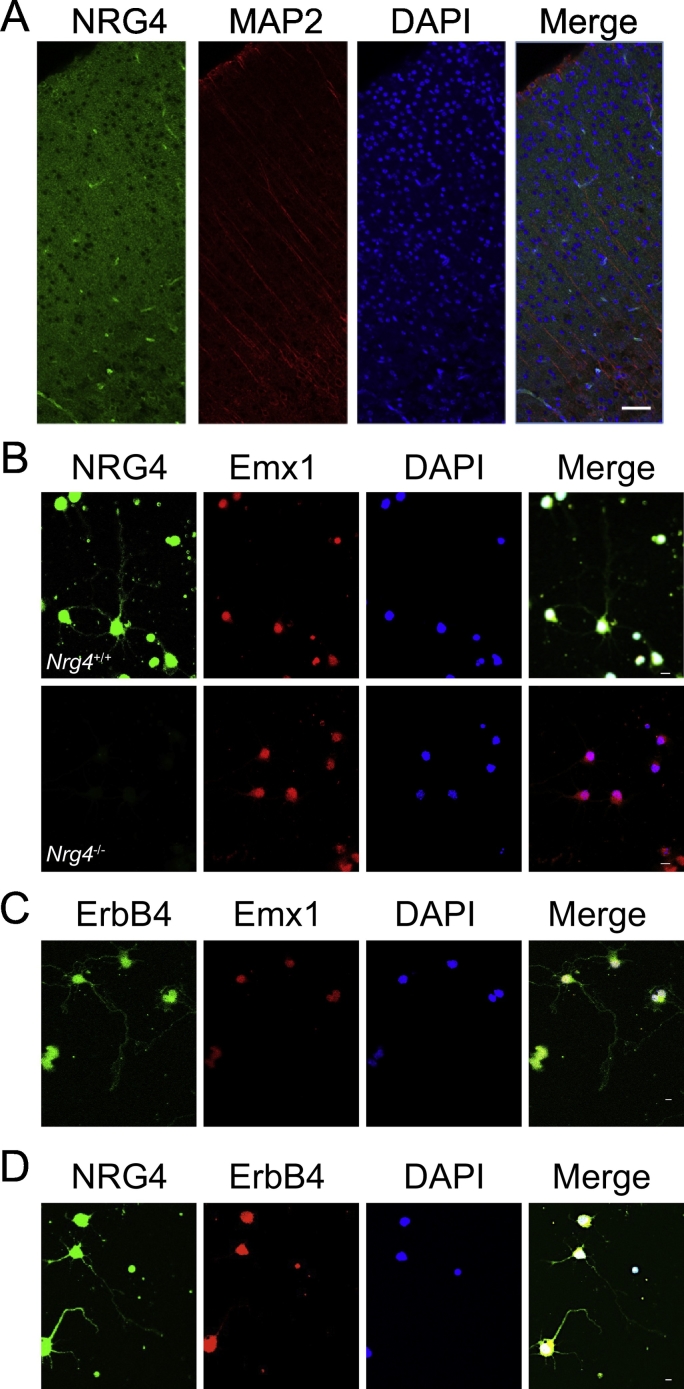
Developing neocortical pyramidal neurons co-express NRG4 and ErbB4. (A) Representative section of the motor neocortex of P10 mice double labelled with antibodies to NRG4 and MAP2. Scale bar = 50 μm. Representative photomicrographs of neocortical neurons of E16 embryos cultured for 3 days and double labelled with antibodies to either NRG4 and Emx1 (B), ErbB4 and Emx1 (C) or NRG4 and ErbB4 (D). To demonstrate the specificity of the anti-NRG4 antibody, cultures were set up from E16 *Nrg4*^−/−^ embryos, which eliminated NRG4 but not Emx1 immunoreactivity (B). Scale bars = 10 μm.

To ascertain the identity of the cells that produce NRG4 in neocortex, we studied NRG4 immunofluorescence in cortical sections and dissociated cortical cultures. In sections of the neocortex of P10 mice, NRG4 immunoreactivity was observed throughout the cortex (Fig. 6A). In dissociated cultures of E16 cortical cultures, double labeling for NRG4 and the pyramidal neuron marker Emx1 revealed that 82.1 ± 2.3% of the Emx1-positive cells were co-labelled with anti-NRG4 after 3 days *in vitro*. NRG4 immunoreactivity was not observed in cultures established from *Nrg4*^−/−^ mice, demonstrating the specificity of the anti-NRG4 antibody (Fig. 6B). The majority of Emx1-positive cells were also co-labelled with antibodies to ErbB4, the principal NRG4 receptor (Fig. 6C). While ErbB4 is abundantly expressed by neocortical and hippocampal GABAergic interneurons (Chen et al., 2010b, Fazzari et al., 2010), lower levels of ErbB4 have been convincingly demonstrated in pyramidal neurons of the neocortex and hippocampus. For example, ErbB4 immunoreactivity is observed in a subset of CamKII-positive cortical pyramidal neurons, staining which is eliminated in mice with conditional deletion of ErbB4 in the brain (Cooper and Koleske, 2014).

The above observations suggest that the majority of embryonic cortical neurons co-express NRG4 and ErbB4. To formally demonstrate co-expression, we double labelled E16 cortical cultures with antibodies to NRG4 and ErbB4 (Fig. 6D). 80.3 ± 5.5% of the cells exhibiting a neuronal morphology were double labelled with these antibodies. This finding increases our confidence that many cortical pyramidal neurons co-express NRG4 and ErbB4, at least in culture, and raises the possibility that NRG4 exerts its effects on pyramidal neurons *in vivo* at least in part by an autocrine/paracrine mechanism. NRG1 autocrine/paracrine signaling has been shown to promote remyelination following peripheral nerve injury (Stassart et al., 2013) and an ErbB4/NRG2 autocrine signaling loop has been demonstrated in inhibitory interneurons (Vullhorst et al., 2015). Neuregulin autocrine signaling has also been implicated outside the nervous system, for example, in promoting the proliferation of certain cancers (Sheng et al., 2010). In future work, it will be interesting to ascertain how the putative NRG4/ErbB4 autocrine loop is regulated in pyramidal neurons, which will provide us with a better understanding of why it is employed in developing brain.

Our demonstration that NRG4 is a major physiologically relevant regulator of the growth and elaboration of pyramidal neuron dendrites in the developing neocortex raises a host of important questions for future work, especially what are behavioural consequences of the major cellular phenotype observed in NRG4-deficient mice and whether NRG4 contributes to the pathogenesis of particular neurological disorders. Given the widespread expression of NRG4 in brain, it will be interesting to investigate whether the lack of NRG4 elsewhere in the brain affects other neurons or circuits and whether axons are affected in addition to dendrites.

## Funding

This work was supported by grant 103852 from the Wellcome Trust.

## Authors' contributions

BP did the culture and Golgi studies, SW did the qPCR and AD supervised the work and wrote the paper.

## Conflicts of interest

The authors declare no conflicts of interest.
